# sRNA-Mediated Regulation of P-Fimbriae Phase Variation in Uropathogenic *Escherichia coli*

**DOI:** 10.1371/journal.ppat.1005109

**Published:** 2015-08-20

**Authors:** Surabhi Khandige, Tina Kronborg, Bernt Eric Uhlin, Jakob Møller-Jensen

**Affiliations:** 1 Department of Biochemistry and Molecular Biology, University of Southern Denmark, Odense, Denmark; 2 Department of Molecular Biology and Laboratory for Molecular Infection Medicine Sweden (MIMS), Umeå Centre for Microbial Research (UCMR), Umeå University, Umeå, Sweden; University of Utah, UNITED STATES

## Abstract

Uropathogenic *Escherichia coli* (UPEC) are capable of occupying physiologically distinct intracellular and extracellular niches within the urinary tract. This feat requires the timely regulation of gene expression and small RNAs (sRNAs) are known to mediate such rapid adjustments in response to changing environmental cues. This study aimed to uncover sRNA-mediated gene regulation in the UPEC strain UTI89, during infection of bladder epithelial cells. Hfq is an RNA chaperone known to facilitate and stabilize sRNA and target mRNA interactions with bacterial cells. The co-immunoprecipitation and high throughput RNA sequencing of Hfq bound sRNAs performed in this study, revealed distinct sRNA profiles in UPEC in the extracellular and intracellular environments. Our findings emphasize the importance of studying regulatory sRNAs in a biologically relevant niche. This strategy also led to the discovery of a novel virulence-associated trans-acting sRNA—PapR. Deletion of *papR* was found to enhance adhesion of UTI89 to both bladder and kidney cell lines in a manner independent of type-1 fimbriae. We demonstrate PapR mediated posttranscriptional repression of the P-fimbriae phase regulator gene *papI* and postulate a role for such regulation in fimbrial cross-talk at the population level in UPEC. Our results further implicate the Leucine responsive protein (LRP) as a transcriptional activator regulating PapR expression. Our study reports, for the first time, a role for sRNAs in regulation of P-fimbriae phase variation and emphasizes the importance of studying pathogenesis-specific sRNAs within a relevant biological niche.

## Author Summary

Recent years have seen an increasing emphasis placed on the role of small RNAs (sRNAs) in the regulation of bacterial gene expression and stress adaptation. The advent of high-throughput sequencing methods has now made it possible to directly monitor the appearance of potentially virulence-associated sRNAs that may contribute to rapid adaptation of the pathogen to a changing environment during infection. Uropathogenic *Escherichia coli* (UPEC) are presumably exposed to a deluge of stimuli from epithelial cell contact, urine and host immune factors and we asked if any regulatory sRNAs would play a role in the transition of UPEC from the extracellular niche to the intracellular one. This study employs co-immunoprecipitation using the RNA chaperone Hfq to identify novel virulence-associated sRNAs in intracellular UPEC, followed by high-throughput RNA-seq. We report the identification of a novel sRNA that we designate PapR (P-fimbriae regulator) and elaborate on this discovery by demonstrating a role for PapR in regulation of P-fimbriae—a UPEC surface virulence factor. The results presented in this study offer new insights into the molecular mechanisms of UPEC pathogenesis and a role for sRNA mediated regulation of virulence factors.

## Introduction

Uropathogenic *E*.*coli* (UPEC) are a group of genetically heterogeneous isolates [1] known to display a complex infection cycle within their host. In the course of acute cystitis, UPEC invade the superficial bladder epithelial cells and replicate in the host cell cytoplasm to form intracellular bacterial communities (IBCs) consisting of coccoid cells arranged in a biofilm-like structure [2]. IBCs subsequently mature into motile rod-shaped bacteria and filaments that are fluxed out of the infected epithelial cell [3, 4]. While a majority of *in vivo*[3, 5] and *in vitro* [6] studies have examined UPEC behaviour in the course of acute urinary bladder infection (cystitis), UPEC are also capable of causing renal tissue infection (pyelonephritis) with more serious clinical implications. This requires timely expression of bacterial virulence genes relevant to different histological niches [7].

UPEC possess an extensive repertoire of secreted virulence factors [8, 9] and surface adhesins that are expressed in a coordinated manner to aid adhesion and invasion of the host epithelium. UPEC isolates are known to encode up to10 fimbrial operons [10] expressing a range of surface adhesins and the best understood among these are type-1 and P-fimbriae. While a synergistic relationship may exist between type-1 and P-fimbriated cells at the population level during ascending urinary tract infection (UTI) [11], it has generally been hypothesized that UPEC predominantly express type-1 fimbriae during infection of the bladder epithelium [12] as opposed to expressing P-fimbriae in the renal environment [13]. The pyelonephritis associated pilus gene cluster, *pap*, encodes for P-fimbriae that recognize the Gal-α(1–4)β-Gal glycosphingolipid moiety on kidney cells via PapG- the adhesive tip of the fimbria [14].

P-fimbrial expression is subject to phase variation involving a reversible epigenetic switch between ON and OFF states which responds to regulator proteins encoded within the operon, PapI and PapB, as well as the global transcription regulators H-NS, cAMP receptor protein (CRP), DNA adenine methylase (Dam) and Leucine responsive protein (LRP) [15]. There are two GATC Dam methylation sites, one distal and one proximal to the main operon promoter (*pBA*), within the regulatory region intergenic to *papI* and *papB*. Reversible binding of LRP and PapI to either one of the two GATC sites allows for Dam-mediated methylation of the unbound exposed site, thereby turning *pap* transcription ON or OFF [16]. Another regulatory protein, PapB, indirectly facilitates phase switching by activating *papI* transcription from the divergent *pI* promoter [17].

Successful bacterial pathogens efficiently adapt to changing environments inside a host during infection. The past few years have seen considerable efforts dedicated towards understanding the survival strategies that intracellular pathogens adopt and the extent to which small regulatory non-coding RNAs are involved. Small regulatory RNAs (sRNAs), with their short half-life, may constitute an efficient means by which pathogens make snap decisions that favour survival in response to environmental stresses during infection [18, 19]. Recent studies [20] have revealed a role for sRNAs in the regulation of virulence factors, particularly surface fimbriae [21, 22], suggesting that rapid sRNA mediated regulation may offer an advantage even in highly regulated fimbrial operon systems.

The majority of the sRNAs discovered in Gram negative bacteria so far are *trans*-acting and exert their function via interaction with Hfq—a homohexameric RNA chaperone protein. Several studies have shown Hfq to be important in the scheme of bacterial virulence [23–25], presumably owing to its role in mediating sRNA-mediated regulation of a wide variety of cellular pathways important to pathogenesis: biofilm formation [26], motility [27] and epithelial cell invasion [28]. Scouting for sRNAs expressed by an intracellular pathogen during infection is fraught with challenges of low RNA yields and although recent years have seen sRNAs consistently reported in the context of infection, few have studied regulation by sRNAs in an intracellular niche [29–31].

This study is based on the premise that the altered transcriptional profile of UPEC during infection and intracellular survival should also be reflected in its sRNA repertoire. In order to identify novel pathogenesis-associated sRNAs expressed by intracellular UPEC infecting human bladder epithelial cells in culture, we adopted an established strategy involving an Hfq-RNA co-immunoprecipitation (co-IP) approach followed by RNA-seq. This study demonstrates that the expressed sRNA profile changes substantially in UPEC upon invasion of bladder cells and further reports the identification of PapR, a novel pathogen specific sRNA enriched during infection that plays a role in P-fimbria phase variation.

## Results

### Deep sequencing of Hfq pull-down RNA from a pathogen reflects its intracellular environment

**Fig 1:**
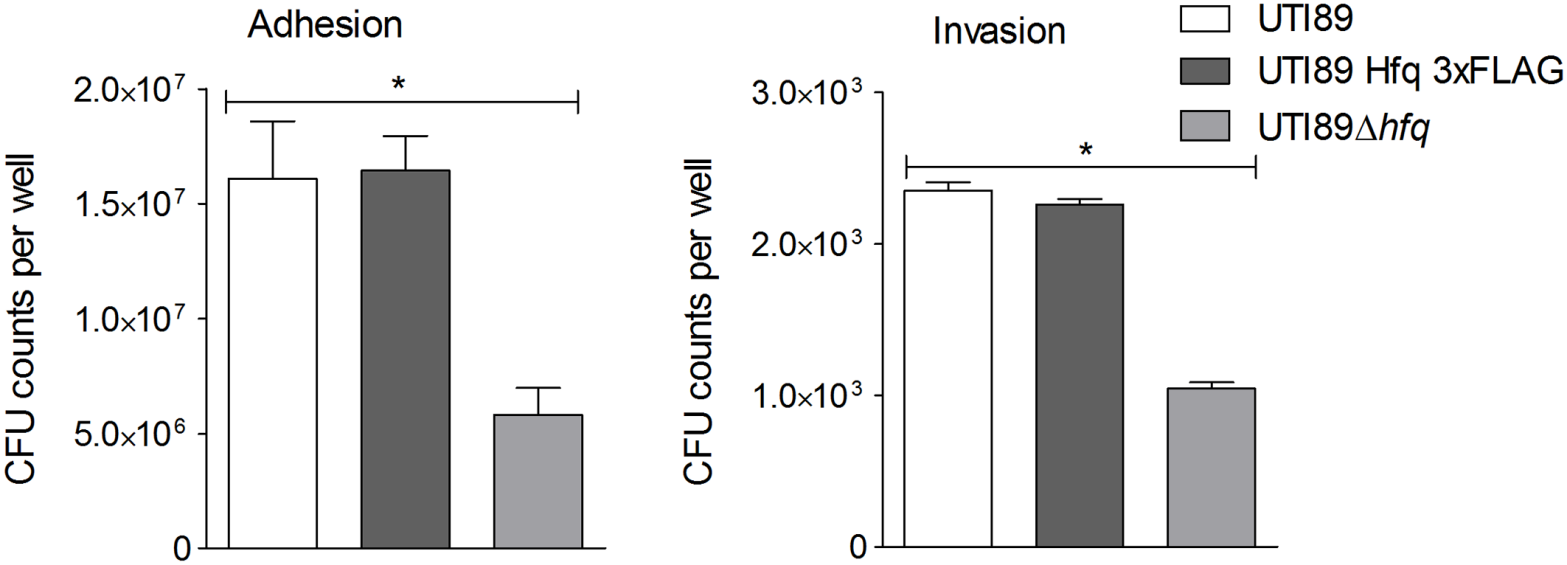
Hfq impact on bladder cell infection. PD07i bladder cells cultured in 24-well plates were infected with UPEC strains cultured overnight at 37°C in LB medium. Mean CFU counts of triplicates from three independent experiments were plotted and standard deviation calculated. UTI89Δ*hfq* showed a statistically significant reduction in adhesion as well as invasion (*p-value <0.05).

This study was aimed at not just obtaining a global snapshot of known Hfq-regulated sRNAs in the course of UPEC infection but, more specifically, identifying novel sRNAs important in the scheme of infection. We constructed a triple FLAG-tagged (3xFLAG) version of *hfq* [32] on the chromosome of UPEC cystitis strain UTI89 [33], to be used for infection of the human bladder epithelial cell line—PD07i [34]. Presence of the Hfq 3xFLAG allowed for efficient co-IP of Hfq and its bound RNA using anti-FLAG monoclonal antibodies. UTI89 expressing Hfq 3xFLAG (UTI89Hfq 3xFLAG) was tested by stationary infection in the bladder cell line and confirmed to match the efficiency of UTI89 expressing untagged Hfq (referred to as UTI89wt from here on) in terms of cell adhesion and invasion capacity (Fig 1), thereby validating its suitability for use in co-IP experiments. The *hfq* deletion mutant UTI89Δ*hfq* displayed a reduced ability to adhere to- and invade bladder epithelial cells (Fig 1).

**Fig 2:**
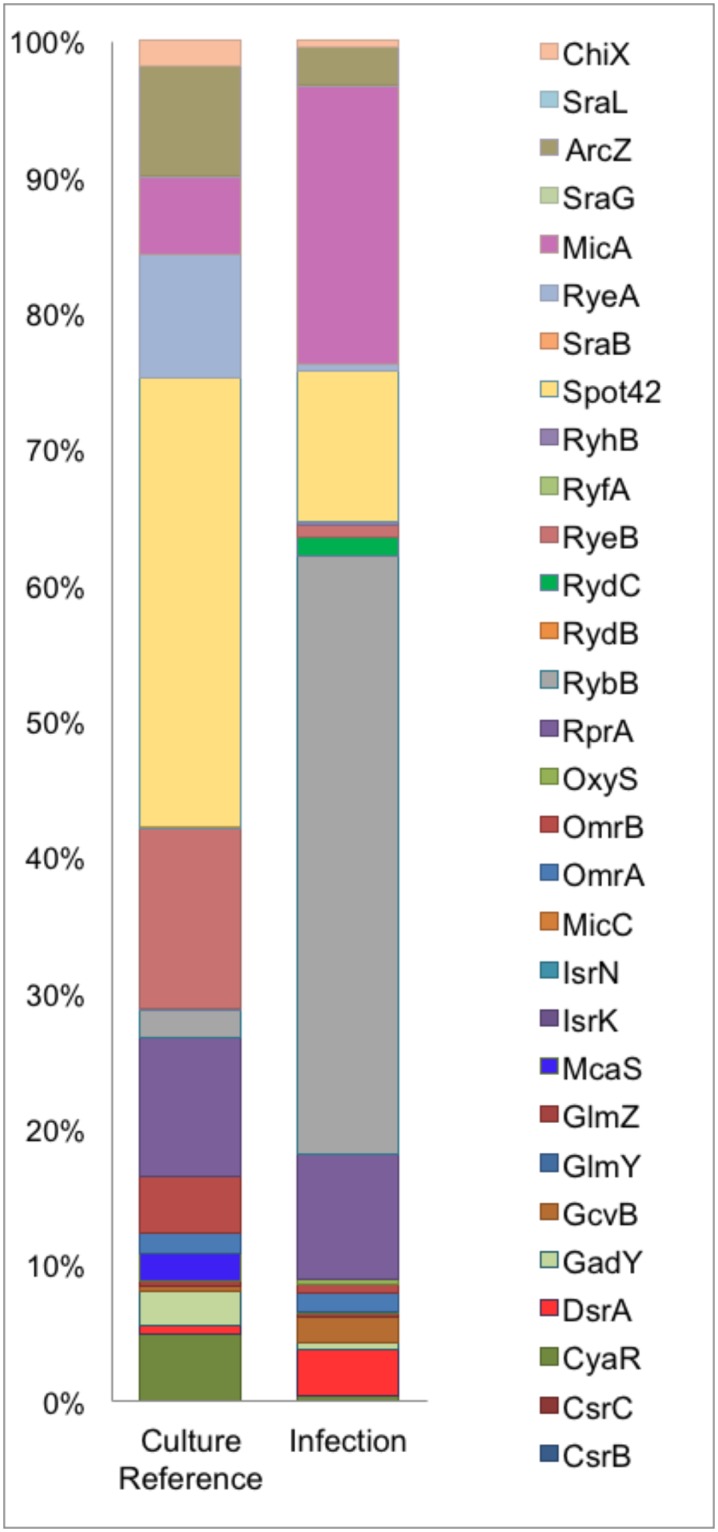
Differential sRNA expression during infection. Graphical representation of percentage coverage of normalized sequence reads from culture reference and infection cDNA libraries corresponding to known sRNAs.

This observation corresponds with other studies showing that Hfq plays a vital role in regulation of virulence-associated genes during infection [35]. Intracellular UTI89Hfq 3xFLAG was harvested from within infected PD07i cells as well as a parallel culture-grown reference and further subjected to Hfq-RNA co-immunoprecipitation and RNA-seq. The distribution and read coverage of sequenced sRNA species from UTI89 during infection and growth in liquid culture were found to be substantially different. This difference likely reflected bacterial adaptation to the intracellular environment. Several known and previously characterized sRNAs were found to be differentially expressed during infection and those most significant have been illustrated in Fig 2.

Our comparison of the sRNA profiles from culture and infection revealed an increase in the expression of MicA and RybB, known negative regulators of outer membrane (OM) proteins [36] in the intracellular compartment along with an observed 2-3-fold decrease in the cDNA sequence reads for their respective targets *ompA* and *ompC* [37, 38]. DsrA and GcvB were also among the upregulated sRNAs during infection while McaS, CyaR, Spot42, RyeA, RyeB and ArcZ were the most down regulated sRNAs during infection. While the overall pattern of sRNAs expressed in the course of our infection study showed a distinct tendency towards functions in intracellular survival and virulence, this profile is representative of the early stages of adhesion and invasion of bladder epithelia.

### Identification of novel sRNAs in the context of UPEC infection

**Fig 3:**
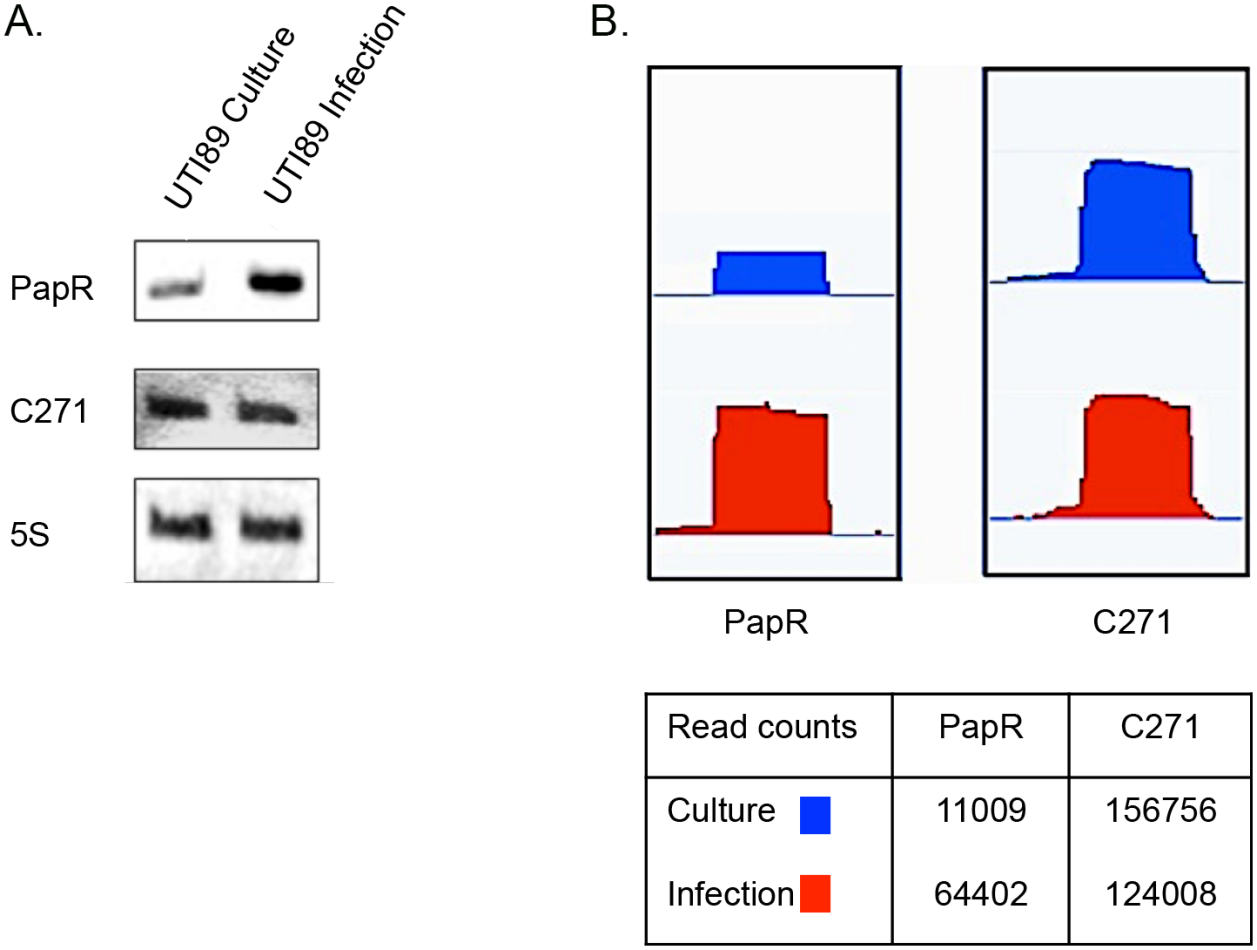
Novel sRNAs detected in UTI89. (A) Northern blots showing transcript levels for PapR and C271. Lanes 1 and 2 represent RNA from culture reference and infection respectively. (B) Graphical output of normalized sequence reads mapping to PapR and C271 in culture reference and infection visualized using Integrated Genome Viewer (Broad Institute). The table below includes normalized total read counts within the PapR and C271 probes.

In order to find novel sRNAs from our high throughput sequencing data we employed SIPHT: an sRNA prediction software [39], and candidates thus identified were mapped to the UTI89 genome (NC_007946.1) and analyzed further. Among the 15 novel candidates tested from *in silico* predictions, two resulted in a stable transcript detectable by Northern blotting and the novel sRNAs were designated PapR and C271 (Fig 3). While C271 levels were mostly unaltered between culture and intracellular growth, PapR showed a ~3 fold increase by Northern blotting (Fig 3A) and close to 6-fold increase in expression in RNA-seq data (Fig 3B) during intracellular growth.

**Fig 4:**
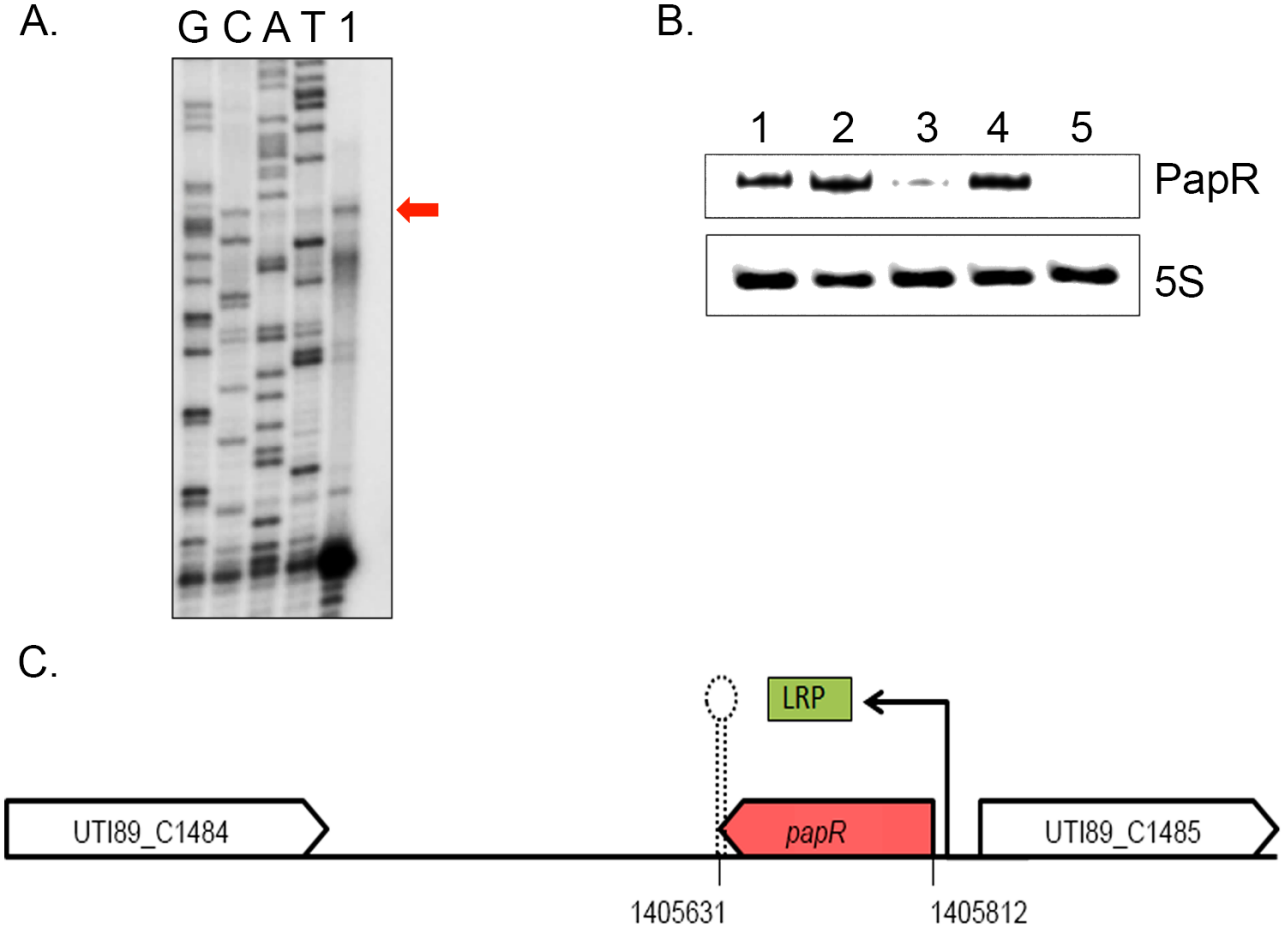
LRP mediated transcriptional activation of PapR. (A) PapR transcriptional start site was mapped using primer extension reaction loaded in the lane marked 1 alongside Sanger sequencing reactions for the four nucleotides represented in lanes GCAT. (B) Northern blotting was used to detect PapR levels in UTI89/pNDM220 (lane 1), UTI89Δ*lrp*/pNDM220 (lane 3) and UTI89Δ*hfq*/pNDM220 (lane 5) with the respective complemented strains UTI89Δ*lrp*/pSKlrp (lane 2) and UTI89Δ*hfq*/pJMJ220 (lane 4), all cultured in LB medium. 5S RNA was used as the internal loading control. (C) Illustration of the genomic context of PapR in the UTI89 genome, drawn to scale. LRP was found to positively regulate *papR* transcription.

Unlike C271, PapR was found in a range of extraintestinal pathogenic *E*. *coli* (ExPEC) strains, enterohemorrhagic *E*. *coli* (EHEC) and *Shigella sp*. but not in *E*. *coli* K-12 (S1 Fig). Interestingly, PapR was not found to be conserved in all UPEC strains, further attesting to the individual genomic variability among UPEC strains that has been reported in the past [1]. Aligned cDNA reads corresponding to both sRNAs were in close agreement to their mapped 5’ termini as confirmed by primer extension (Fig 4A, S2 Fig). Owing to the differential expression observed during infection, subsequent efforts reported in this study were directed towards the elucidation of PapR function. MFold RNA secondary structure predictions [40] suggest that PapR contains a Rho-independent terminator along with AU-rich single-stranded regions, which could potentially interact with Hfq (S3 Fig).

Northern blot analysis revealed that PapR levels were drastically reduced in UTI89Δ*hfq* (Fig 4B), indicating that the expression or stability of PapR was dependent on functional Hfq.

Examination of the *papR* promoter region revealed a partial LRP consensus binding site composed of flanking CAG/CTG triplets and an intervening AT-rich sequence at ~83 nt upstream of the mapped transcription start site. We probed UTI89Δ*lrp* for PapR and found that deletion of *lrp* abrogated PapR expression, thereby identifying LRP as a transcriptional activator of PapR expression. The expression of PapR was correspondingly restored to wildtype levels in the Δ*lrp* and Δ*hfq* complemented strains (Fig 4B and 4C).

### PapR regulates the expression of P-fimbria

**Fig 5:**
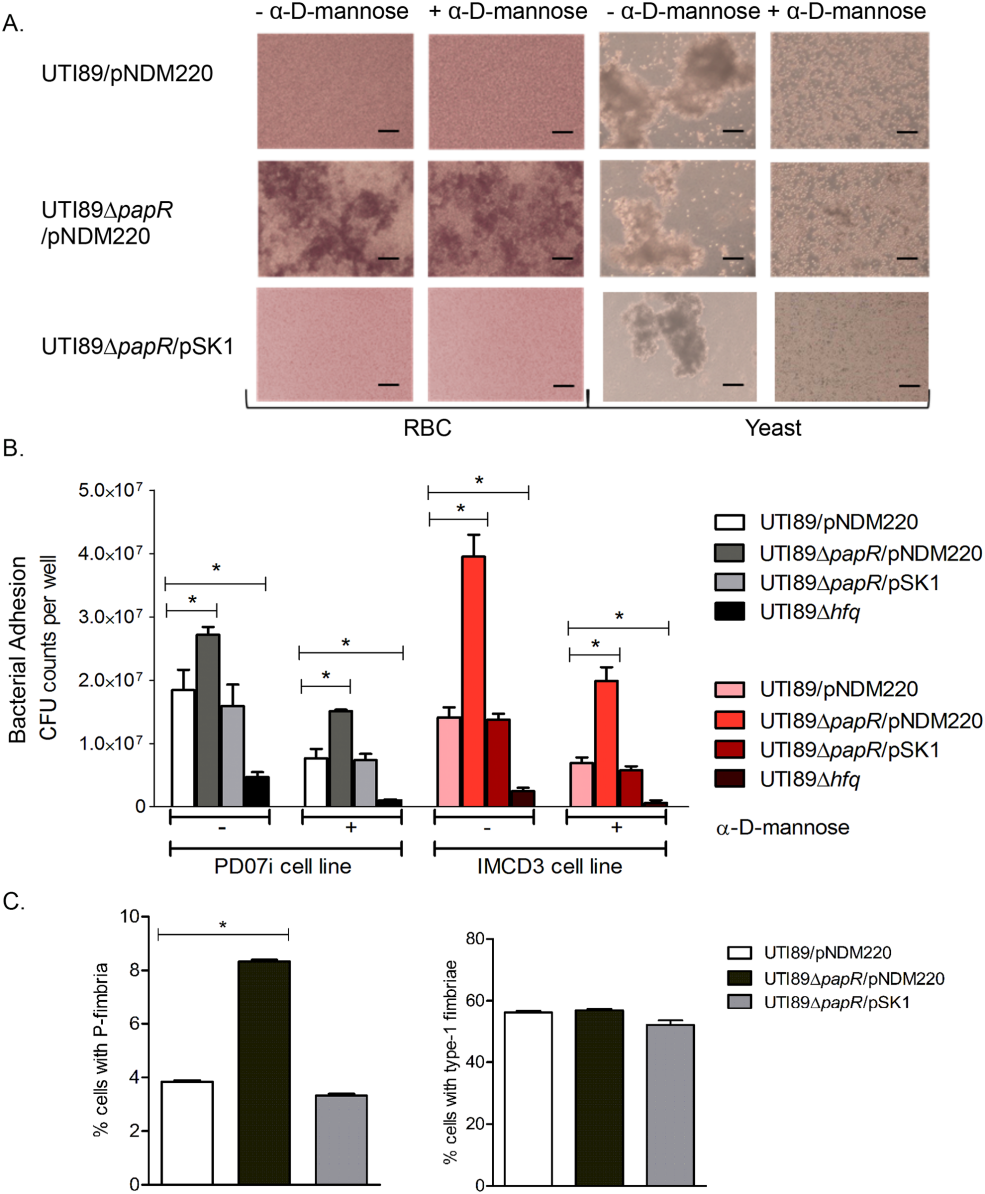
Characterization of PapR function. (A) Heme- and yeast cell agglutination assays performed with UTI89/pNDM220, UTI89Δ*papR*/pNDM220 and UTI89Δ*papR*/pSK1 with and without the addition of α-D-mannose. Scale bars set at 50 μm. (B) PD07i bladder cells and IMCD3 kidney collecting duct cells cultured in 24-well plates were infected with UTI89/pNDM220, UTI89Δ*papR*/pNDM220, UTI89Δ*papR*/pSK1 and UTI89Δ*hfq*. Strains were either left untreated (-) or treated (+) with 3% α-D-mannose and used for infection. Bacterial adhesion was assessed by calculating mean CFU counts from three independent experiments. Statistical significance was calculated using Students t-test (*p-value <0.05). (C) Flow cytometry of UTI89/pNDM220, UTI89Δ*papR*/pNDM220 and UTI89Δ*papR*/pSK1 grown overnight in LB medium and immunolabelled with α-PapA and α-Fim antibodies was used to detect the extent of P- and type-1 fimbriated cells respectively. Mean fluorescence from three independent experiments was plotted along with the standard deviations. Statistical significance was calculated using Students t-test (*p-value <0.05).

In order to characterize PapR function, we created a *papR* deletion strain UTI89Δ*papR* and a low-copy number inducible PapR expression plasmid, pSK1, which allows for complementation of phenotypes associated with *papR* deletion. The P_A1/O4/O3_ promoter driving *papR* expression from pSK1 was induced by the addition of 1mM IPTG (isopropyl-β-d-thiogalactopyranoside). We found that deletion of *papR* did not affect UTI89 growth rate in LB medium or Epilife cell culture medium (S4 Fig). Using CopraRNA [41], we performed an *in silico* mRNA target prediction and found *papI* mRNA to be among the predicted targets of PapR mediated regulation. Alternative *in silico* predicted mRNA targets are listed in S1 Table. In an attempt to further investigate the potential involvement of PapR in regulation of P-fimbrial biogenesis, agglutination assays with yeast cells and human erythrocytes (RBC) were performed. Bacterial cultures grown statically in liquid media are known to show a propensity towards expression of type-1 fimbriae and this preference switches to P-fimbriae during growth on solid surfaces [42]. In light of this, we tested UTI89/pNDM220 (vector control), UTI89Δ*papR*/pNDM220 and UTI89Δ*papR*/pSK1 for P-fimbriation in statically grown overnight LB cultures so as to enhance any contrasts in agglutination phenotypes between the UTI89 with and without PapR. While UTI89/pNDM220 displayed mannose sensitive yeast agglutination, UTI89Δ*papR*/pNDM220 displayed mannose resistant agglutination to a small extent in yeast cells (Fig 5A right) and to a much greater extent in RBCs (Fig 5A left). The mannose resistant agglutination phenotype was lost in the complemented strain UTI89Δ*papR*/pSK1 (Fig 5A bottom row), suggesting that PapR negatively regulates expression of a mannose resistant fimbrial type like P-fimbriae. The role of PapR as a mediator regulating mannose resistant surface adhesins was subsequently investigated in an *in vitro* infection assay using human cell lines derived from the urinary bladder (PD07i) and inner medullary collect ducts of kidney (IMCD3). Bacterial adhesion to both cell lines was tested with untreated and α-D-mannose pre-treated strains in parallel in order to not only estimate the influence of PapR on cell adhesion but also clearly demonstrate type-1 fimbriae independent adhesion. In concurrence with our observations from the agglutination assay, UTI89Δ*papR*/pNDM220 demonstrated increased adhesion (*p*-*value* <0.05) to bladder and kidney epithelial cells in a mannose resistant manner (Fig 5B). As observed with the agglutination assay previously, this phenotype was absent in the UTI89Δ*papR*/pSK1 complemented strain. While PapR was found to have no significant bearing on the pathogen’s ability to invade bladder cells, the IMCD3 kidney cell line however, demonstrated a clear increase in adhesion as well as invasion when infected with UTI89Δ*papR* (S5 Fig).

The results obtained from agglutination- and adhesion assays indicated that deletion of *papR* resulted in an increase in mannose-resistant surface fimbriae such as P-fimbriae, at least at the population level. In order to examine the influence exerted by PapR at the single cell level, flow cytometric analysis using UTI89 strains immunostained for P- and type-1 fimbriae was performed. Strains were grown statically overnight in LB medium and immunolabelled in parallel with either anti-PapA or anti-Fim antibodies, to reveal a mixed population with cells expressing type-1 as well as cells expressing P-fimbriae on their surface. Fluorescence microscopy imaging as well as Western blotting of cultures grown statically overnight in LB medium was performed to confirm antibody labelling specificity for both anti-PapA and anti-Fim antibodies (S6 Fig). While UTI89/pNDM220, UTI89Δ*papR*/pNDM220 and UTI89Δ*papR*/pSK1 all demonstrated simultaneous expression of both P- and type-1 fimbriae at the population level (Fig 5C); P-fimbriated cells composed a much smaller fraction of the population in both UTI89/pNDM220 and UTI89Δ*papR*/pSK1. Deletion of *papR* resulted in a doubling of the fraction of P-fimbriated cells (*p-value <0.05), whereas in contrast, the fraction of type-1 expressing cells appeared to be unaffected by *papR* deletion.

### PapR based repression of P-fimbrial phase variation via *papI* targeting

**Fig 6:**
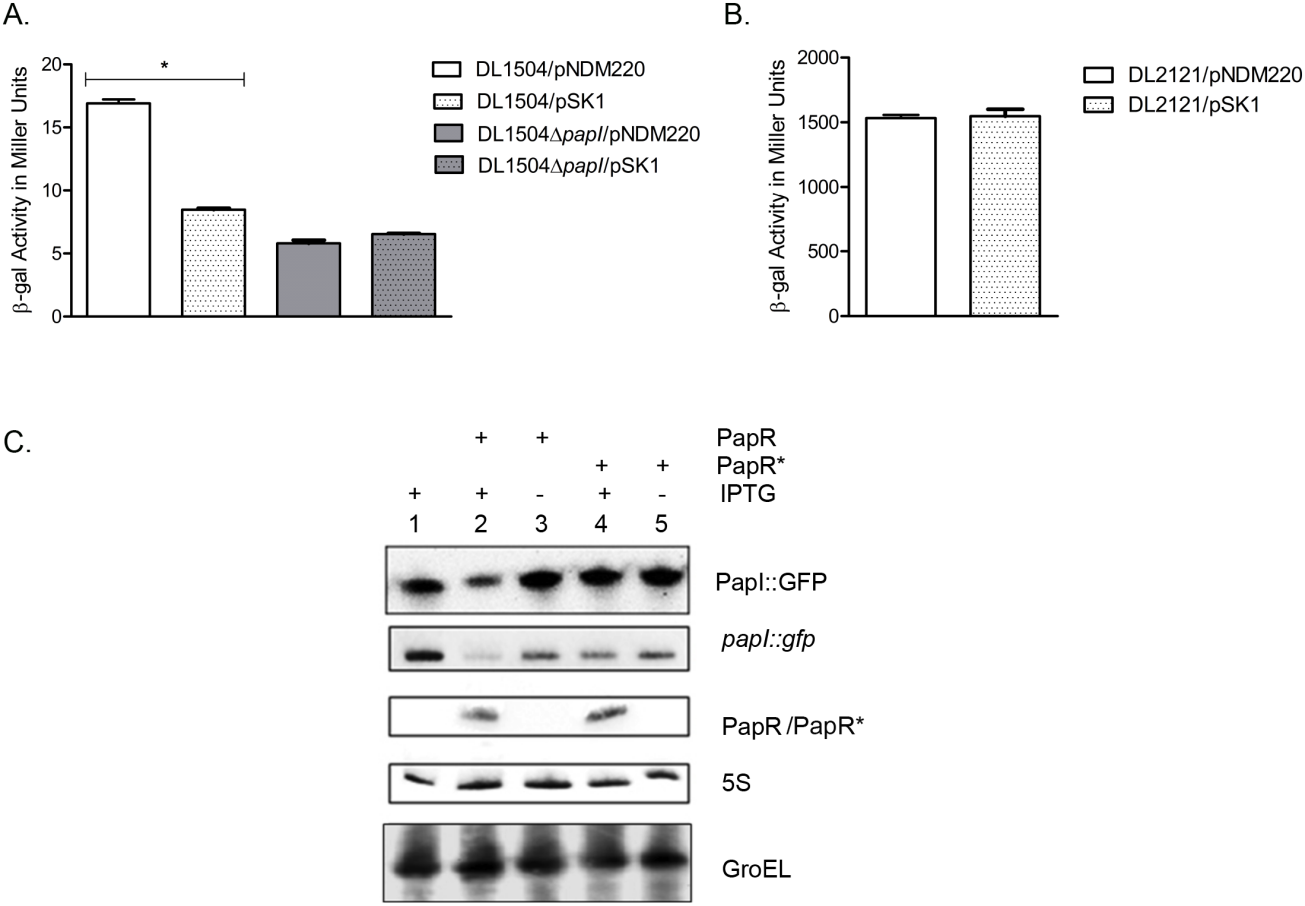
PapR mediated *papI* repression. (A) β-galactosidase assays were performed with DL1504 strains with and without *papI*. Induction of PapR using 1mM IPTG resulted in a 50% reduction in β-galactosidase activity in DL1504 (*p-value <0.05) but not DL1504Δ*papI*. Mean values from three independent experiments were plotted and standard deviations calculated. (B) PapR was induced with 1 mM IPTG in DL2121 strains and mean values of β-galactosidase activity measured in Miller units were plotted. (C) Validation of *papI* as a PapR target was performed using Top10/pSKpapI/pNDM220 (lane 1), Top10/pSKpapI/pSK1 (lanes 2–3) and Top10/pSKpapI/pSK1* (lanes 4–5). Strains were grown with (lanes 1, 2 and 4) and without (lanes 3 and 5) the presence of 1 mM IPTG to induce PapR from pSK1 and PapR* from pSK1*. Northern blot detection of PapR, PapR* and *papI*::*gfp* levels was carried out in parallel with Western blot detection of PapI::GFP. GroEL and 5S were used as internal controls in the Western and Northern blots respectively.

The *pap* gene cluster is present as a single copy in the UTI89 genome [43] unlike some other UPEC strains, making it a more straightforward subject of study. Expression of the *pap* gene cluster is regulated on multiple levels by different but interconnected global regulators along with two regulatory proteins encoded within the *pap* gene cluster—PapI and PapB. The regulatory genes *papI* and *papB* are transcribed from two divergent promoters—*pBA* and *pI*. Transcription from the *pBA* promoter results in a primary transcript which is quickly cleaved by RNase E into separate *papB* and *papA* transcripts which differ in their stability as RNA transcripts [44]. In order to ascertain the *in vivo* regulatory effect on *papI* mediated by PapR- a regulatory sRNA located outside the *pap*-associated pathogenicity island of UTI89, we chose to adopt a well-established approach using *pap-lac* operon reporter fusions [16, 45, 46]. We performed β-galactosidase assays by ectopically expressing PapR from pSK1 in *E*. *coli* K-12 derived strains, DL1504 and DL2121 that lack an endogenous *pap* gene cluster but are genetically modified to carry a *lac* operon fusion in the chromosome. Details of DL1504 and DL2121fusion constructs have been illustrated by Braaten et.al [16] and Nou et.al. [47] respectively. These strains contain a chromosomal insertion of the entire *papI* and *papB* genes with their native divergent promoters and the intergenic *pap* regulatory region between them, with a *papB-lacZ* operon fusion. In addition, the *papB* promoter region in the DL2121 strain carries a mutation in the LRP binding site #3 rendering it independent of PapI mediated phase regulation. We found that the DL1504 strain with the pNDM220 empty vector showed the same level of β- galactosidase activity (measured at 17 Miller units) as the parental DL1504 without plasmid and that upon induction of PapR expression with 1mM IPTG the β-galactosidase activity of DL1504/pSK1 was reduced by half. These findings were compounded by the lack of any discernible change in β-galactosidase activity both in DL1504*ΔpapI* and DL2121 (Fig 6A and 6B), indicating that regulation of P-fimbriae expression by PapR was more likely to be mediated at the level of *papI* mRNA. PapI is known to play a role in the phase ON/OFF transition of the *pap* operon by regulating the alternation of LRP binding from sites 1, 2, 3(OFF) to sites 4, 5, 6 (ON) [48].

*In silico* predictions revealed a region of near-perfect complementarity spanning 22 nucleotides in PapR and in *papI* mRNA, including most of the AU rich regions upstream of the PapR terminator (S3 Fig). The putative region of interaction between *papI* mRNA and *papR* was predicted to be 74–96 nucleotides downstream of the *papI* translation start site and thus was suggestive of PapR regulating *papI* mRNA by means of destabilization of the mRNA [49, 50].

In order to confirm that down regulation of P-fimbriae was mediated through direct base pairing between PapR and *papI* mRNA, we constructed a plasmid vector pSK1* to express an altered PapR sRNA (PapR*) with an inverted terminator stem region, thus abolishing base pairing with the target mRNA while ensuring that the PapR* sRNA would not suffer secondary structural perturbations. As a reporter we constructed a *papI*::*gfp* translational fusion as devised previously by Urban and Vogel [51]. Owing to the position of the predicted region of interaction inside the *papI* ORF, the entire length of the gene in addition to a short upstream region at the 5’ end was cloned into a low copy number vector—pXG10, with a constitutively active λ_PL_ promoter. PapR and PapR* sRNAs were expressed from pSK1 and pSK1* respectively using 1mM IPTG induction. These assays were performed in an *E*.*coli* K-12 background without endogenous *pap* gene cluster or *papR*. All transformants were grown aerobically to exponential phase in LB medium and cultured for an additional 30 mins with or without IPTG, after which cells were harvested for total RNA isolation for Northern blotting and whole cell lysates for Western blotting. By detecting RNA levels of *papI*::*gfp* and *papR* in conjunction with protein levels of PapI::GFP, a composite view of PapR-mediated post-transcriptional repression of *papI* could be visualized. Fig 6C shows that PapR and PapR* were synthesized at similar levels upon IPTG induction, but only PapR leads to a reduction in the *papI*::*gfp* mRNA level (~3 fold) and GFP protein level (~2 fold). The absence of any PapR*-mediated regulation supports the suggested requirement for a direct base pairing interaction between PapR and its *papI* mRNA target.

## Discussion

The role played by sRNAs in making rapid cellular decisions and regulating transcription in pathogenic bacteria, although currently underrepresented in the literature, could be a substantial one [52, 53]. In this study, the uropathogenic *E*. *coli* strain UTI89 harvested from within infected bladder epithelial cells was investigated in order to identify novel virulence-associated Hfq-interacting sRNAs. We report the discovery of two sRNAs conserved among related ExPEC strains which were designated PapR and C271.

Bacterial sRNAs are broadly categorized as *cis-* and *trans-*encoded regulators of gene expression. In Gram negative bacteria, a vast majority of *trans-*encoded sRNAs are known to depend on Hfq- a global RNA chaperone, for structural stability and regulatory function [54]. Bacterial sRNAs are known to demonstrate target mRNA repression either by obscuring the ribosome binding site to prevent translation or by mediating target mRNA degradation. Conversely, sRNAs are also known to function as translational activators and in both instances, a region of sequence complementarity is required for this sRNA-mRNA interaction and Hfq enables this interaction [55, 56].

Overall, our survey of UTI89 sRNA profiles during infection and *in vitro* culture displayed a pronounced tendency towards stress adaptation in the intracellular environment. During infection, UTI89 showed increased expression of sRNAs responding to envelope stress—MicA, RybB and DsrA. It has been known for some time, that cases of acute UPEC-mediated cystitis are associated with the development of tightly clustered intracellular bacterial communities (IBCs) [5] and that IBC development is crucial to the later stages that manifest in the scheme of UPEC infection [57]. Outer membrane (OM) proteins, particularly OmpA, have been shown to be critical for IBC maturation later in infection but not essential during the early stages of adhesion and invasion of bladder epithelia [58]. In this context, MicA and RybB may facilitate rapid down regulation of OM proteins in the initial phase of infection, only to enable *omp* expression when required again during the later IBC stage. DsrA, which was also increased in infection, is a known positive regulator of RpoS [59]- an alternate sigma factor vital to stress response that has been implicated in UPEC virulence and intracellular survival in the urinary bladder [35, 60]. Another positive regulator of RpoS activity [61], GcvB RNA, was also found to be induced in intracellular UPEC. GcvB is further known to regulate amino acid metabolism [62], the transcriptional regulator of curli synthesis CsgD [63] and the PhoP/Q two component system [64]. Interestingly, GcvB is also a known negative regulator of LRP [65], a transcription factor that we show regulates PapR expression in UTI89. Intracellular pathogens adopt sRNA mediated regulation as part of a defence strategy that aids survival inside a hostile host environment as well as an important part of an offensive response the pathogen mounts against its host [66, 67]. Chao et.al. [68] demonstrated the changing sRNA profile in an LB culture of *Salmonella* over time and in doing so, emphasized the dynamic nature of sRNA expression and the rapid regulation it mediates. At a single time point, our sRNA profile during infection is more likely to be a snapshot view of early adaptations to intracellular survival within bladder epithelial cells.

UPEC are known to possess a variety of virulence factors ranging from adhesive fimbriae on the cell surface to secreted toxins that render them potent pathogens. Fimbrial adhesion to the host epithelium is an important first step in early infection and some UPEC strains have been reported to possess as many as ten putative fimbrial operons [69], and of these, type-1 and P-fimbriae are among the better characterized. Pichon et.al. [20] recently reported several virulence-associated antisense RNAs (asRNA) in the UPEC strain 536 grown in LB medium, one of which was FimR, a regulator of type-1 fimbriae. Despite the *fim* operon being highly conserved across UPEC strains, our RNA-seq data did not reveal read counts corresponding to FimR. This could in turn reflect back to the fact that our study involved an Hfq co-IP which might result in a failure to represent asRNAs in the samples since they are less likely to be Hfq dependent [55]. We suggest that this should be considered as a potential limitation in studies employing a strategy solely involving Hfq co-IP.

Studies have shown that UPEC infection of the urinary bladder is predominantly associated with the expression of type-1 fimbriae [70, 71]. However, agglutination of erythrocytes by UTI89Δ*papR* in an α-D-mannose resistant manner and a lack of *in silico* predicted targets from the *fim* operon, decidedly pointed away from any direct role for PapR in expression of mannose sensitive type-1 fimbriae. Concurrently, by performing parallel adhesion assays where strains were untreated or treated with α-D-mannose prior to infection, it was possible to provide clear evidence of mannose resistant surface fimbriae being responsible for the elevated adhesion observed for UTI89Δ*papR*. Infection was carried out in two human cell lines derived from the urinary bladder and inner medullary collecting ducts of the kidney, representing two distinct pathological niches UPEC are known to occupy. Although UTI89Δ*papR* demonstrated significantly improved adhesion in both cell lines, this effect was greater in the kidney derived cell line. The kidney cell line also displayed a significant increase in UTI89Δ*papR* invasion when compared to UTI89/pNDM220, confirming that the nature of surface fimbriae could influence UPEC tissue tropism. However, this improved adhesion was not followed by a subsequent increase in invasion in the bladder derived cell line; a phenomenon that has been previously reported in relation to P-fimbriae and *in vitro* infection of bladder epithelial cell lines [72]. A reduced propensity towards epithelial cell adhesion and invasion was observed for UTI89Δ*hfq* in accordance with previous reports [35]. It was however interesting to note that α-D-mannose treatment further debilitated UTI89Δ*hfq*, perhaps indicating that while Hfq does not influence expression of type-1 fimbriae in UTI89, it could have bearing on alternative virulence factors critical for bladder cell adhesion. Since *fim* and *pap* operon deletion mutants were not included in the infection assays, improved epithelial cell adhesion of UTI89Δ*papR* in the infection assays could not be attributed with certainty to P-fimbriae but rather just to a mannose resistant fimbrial type, While the agglutination and adhesion assays provide hints as to the nature of surface fimbriae expressed at the population level, by fimbriae-specific immunolabelling and flow cytometry we were able to examine single cells to demonstrate a greater fraction of P-fimbriated cells in the PapR null mutant when compared to UTI89wt. Our findings with flow cytometry revealed the presence of both type-1 fimbriated and P-fimbriated bacteria in the population. This could indicate that decisions favouring one type of surface adhesin over another could be made on a population scale, by elevating synthesis of mediators like PapR that would tip the scales in favour of a particular adhesin: type-1 fimbriae [11, 73]. The expression of a particular fimbrial variant is dependent on the tissue niche UPEC occupy and studies have shown that expression of type-1 fimbriae is favoured during infection of bladder epithelial cells since they are more rigid and resistant to urine flow [74] and expression of P-fimbriae is favoured when the pathogen ascends to the renal pelvis and infects the kidney [13]. Our observation that mean fluorescence did not significantly vary across strains labelled with α-PapA antibody, further supports the idea that PapR expression is more likely to alter the ratio of P-fimbriated cells in the population rather than the extent of P-fimbriation on an individual cell. This could indicate that there might be selective expression of PapR within population subgroups in response to as yet unknown environmental cues.

Despite previous reports suggesting minimal involvement of P-fimbriae in UPEC infections [13], Buckles et.al. [75] recently showed that P-fimbriae fulfil Koch’s postulates as a bona fide virulence factor and form part of the pathogenesis repertoire even in UPEC strains causing acute cystitis. While the precise advantage conferred by P-fimbriae to UPEC in the course of infection is still unclear, P-fimbriae have been purported to aid ascension of the pathogen from the bladder and establishment of infection in the kidney [75, 76] as well as function as an immunomodulant influencing the immune responses within a local niche [77–79].

**Fig 7:**
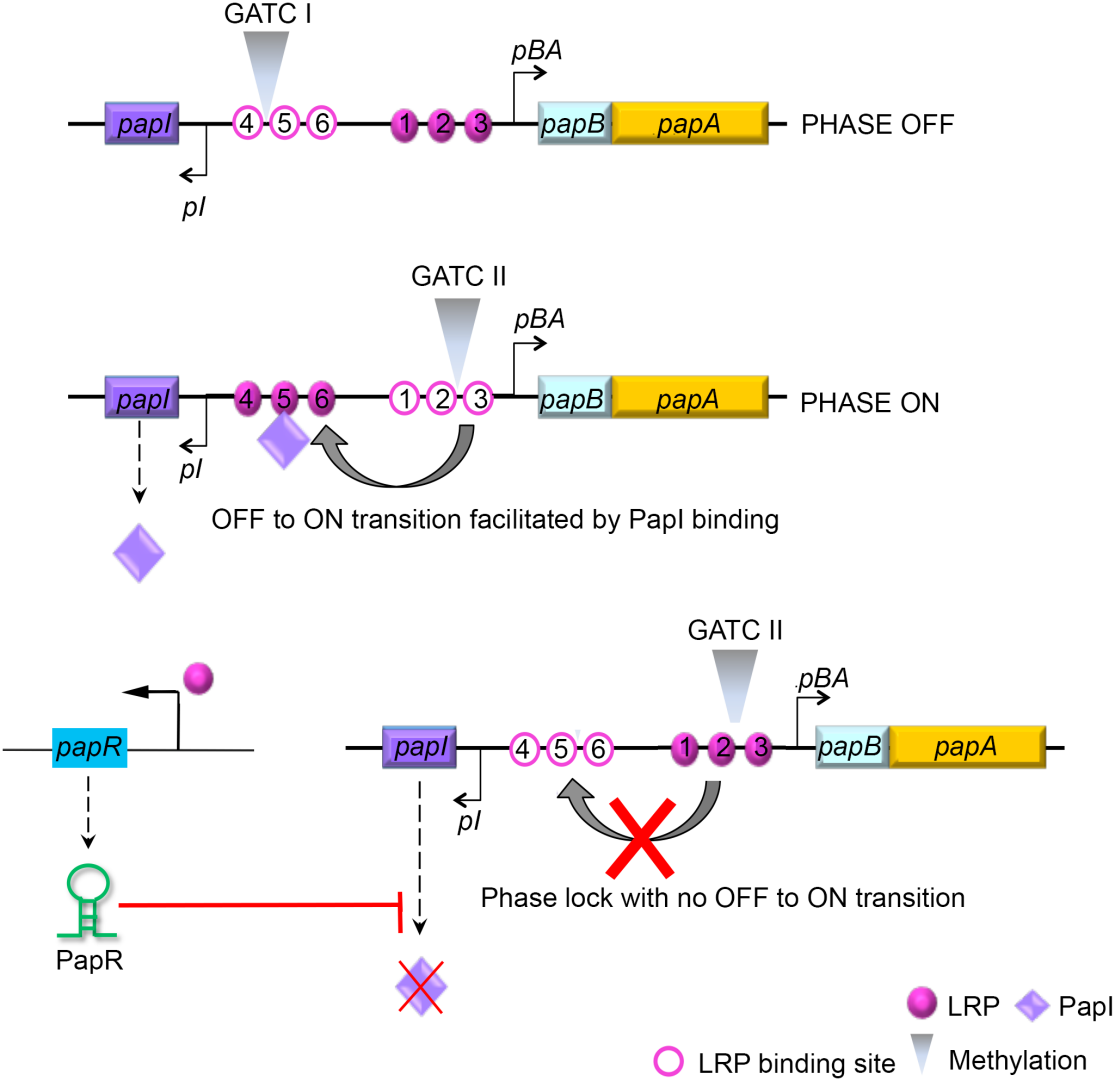
Model illustrating PapR-mediated modulation of P-fimbriae phase variation. The *pap* gene cluster encodes two regulatory proteins, PapI and PapB, that work in concert with other global regulators such as LRP, H-NS and Dam methylase to control P-fimbrial phase variation between the OFF and ON states. LRP mediated transcriptional activation of PapR sRNA results in the degradation of *papI* mRNA. An absence of functional PapI results in a failure to switch from an OFF to an ON phase, and a failure therein to express P-fimbriae on the surface.

Pathogens are able to rapidly perceive environmental cues and correspondingly modulate their choice of surface adhesins by a means of phase variation, which is essentially believed to be an immune evasion strategy as well as an adaptive response to boost the odds of bacterial survival in the face of a changing environment [80]. Especially in pathogenic bacteria, ON/OFF phase variation of surface adhesins could be key to expressing the best suited adhesin for the target tissue, in a sequential manner [81]. The presence of an LRP consensus sequence upstream of the PapR transcription start site along with the abrogation of *papR* transcription in an *lrp* null mutant identifies LRP as a transcriptional activator of PapR synthesis. This finding adds an additional layer of complexity and implies a dual role for LRP in P-fimbriae phase switching under the conditions when bacteria are localized in host cells. A summarizing model illustrating the roles we now have identified for PapR and Lrp in modulation of P-fimbriae phase variation is shown in Fig 7. LRP is a global regulator known to directly influence both type 1 and P-fimbriae phase variation in *E*.*coli*. Regulation of PapR could represent an additional indirect means of LRP involvement in type 1and P-fimbrial crosstalk.

Apart from the global regulators cAMP-CAP, H-NS, Dam methylase and LRP, the *pap* operon is regulated by proteins encoded within the gene cluster, PapI and PapB. PapI is known to be involved in ON/OFF phase transition of P-fimbria by interacting with the *pap* DNA-LRP complex [82, 83]. This PapI interaction results in translocation of the complex occupying LRP binding sites #1–3 and blocking *pBA* transcription, to LRP binding sites #4–6, resulting in activation of the *pBA* promoter. Results ensuing from the β-galactosidase assays using strains with a functional *papI* (DL1504), *papI* null strain (DL1504Δ*papI*) and *papI* independent expression system (DL2121), all indicated that PapR targeted *papI* mRNA and thereby regulated P-fimbrial phase variation. A GFP reporter system was used to not only confirm direct interaction between PapR and its target *papI* mRNA, but also to identify the region of interaction by employing the pSK1* plasmid construct with an inverted terminal stem loop region. The lack of any reduction in the levels of *papI*::*gfp* mRNA as a consequence of PapR* expression from pSK1* plasmid, confirms the *in silico* predicted region of interaction to hold true.

PapR overall expression levels being two fold higher during infection than in the culture grown reference condition, could reflect a need to support type-1 fimbriae expression (preferable during bladder cell invasion) while simultaneously repressing P-fimbriae expression, when infecting bladder epithelial cells. Owing to early experimental evidence suggesting involvement of PapR in P-fimbrial regulation, no alternative mRNA targets from *in silico* predictions were explored in this study. This however, does not preclude the possibility that PapR might well act on multiple fronts in UPEC.

PapR sRNA represents a new principal addition to the repertoire of macromolecules involved in *pap* regulation, presumably promoting the advantage of a rapid ON/OFF switch favouring the best-suited surface adhesin at the time within at least part of the population, and thereby enhancing the odds of the pathogen’s survival within its specific tissue niche. A few recent studies have reported sRNA-mediated regulation of surface fimbriae in pathogenic bacteria [20, 84] further substantiating our hypothesis regarding the value of sRNA mediated modulation of gene expression even in an already heavily regulated system.

Survival of a pathogen and success in infection, hinges on its ability to efficiently sense signals from the host environment and make suitable adaptations [85] including a modulation of its own surface in accordance with the surrounding niche. In this work we took advantage of high throughput sequencing technology that allows analyses of low input starting material in order to explore pathogen behaviour in its biologically relevant niche. There has been a growing role attributed to sRNAs in the context of bacterial pathogenesis as a versatile regulatory device in control of bacterial virulence strategies. This study revealed that intracellular pathogens express their own unique sRNAs as part of their infection armament. The discovery of PapR, and C271 specifically in pathogenic *E*. *coli* and their expression in the context of infection, should prompt additional efforts to clarify the complete sRNA repertoire that such pathogens possess.

## Materials and Methods

### Bacterial strains and growth conditions

A cystitis-derived isolate *E*. *coli* UTI89 with serotype O18:K1:H7 was used for RNA sequencing and strain constructions (S2 Table). *E*. *coli* K-12 derived DL1504 and DL2121 were used for β-galactosidase assays and Top10 was used for validation of sRNA target interaction. UTI89 harvested for RNA-seq was obtained from infection and culture reference involving overnight lysogeny broth (LB) culture followed by 3 hours of static growth at 37° C in Epilife cell culture media to replicate infection conditions. Strains for other experiments were grown in LB broth supplemented with 100 μg/ml ampicillin, 40 μg/ml chloramphenicol (Cml) and 40 μg/ml kanamycin (Kan) as required. Expression was induced from the P_A1/O4/O3_ promoter with 1 mM isopropyl-β-d-thiogalactopyranoside (IPTG).

The deletion strains UTI89Δ*hfq*, UTI89Δ*papR* and UTI89Δ*lrp* were made using pKD3/pKD4 plasmid templates to amplify Cml and Kan resistance cassettes respectively as described by Datsenko and Wanner [86]. Insertion of Cml cassette was confirmed using primers JMJ99 and JMJ100 and Kan cassette using primers JMJ71 and JMJ72. UTI89Hfq 3xFLAG was constructed using primers JMJ155 and JMJ156 and pSUB11 plasmid template as detailed by Uzzau *et*.*al*.[32]. Plasmid pSK1 (JMJ835+JMJ836), pSK1* (JMJ835+JMJ645) and pSKlrp (JMJ939+JMJ940) were derived by cloning native PapR, PapR* with an inverted terminator stem region and *lrp* respectively into the pNDM220 vector as described by Boysen *et*.*al*.[87] and constructs were confirmed using JMJ207+JMJ221. Plasmid pSKpapI was derived from pXG-10 plasmid using plasmids JMJ641+JMJ642 to construct a *papI*::*gfp* translational fusion and confirmed using JMJ732+JMJ733. All relevant primer pairs are listed in S3 Table.

### Stationary infection

PD07i bladder epithelial cells kindly provided by David Klumpp [34] were cultured and maintained in Epilife medium supplemented with human keratinocyte growth serum (Invitrogen) as described previously [6]. Human kidney derived inner medullary collecting duct IMCD3 cells were generously donated by Per Svenningsen and cultured in DMEM:F12 nutrient mix cell culture media (Life Technologies) supplemented with 10% FBS. PD07i and IMCD3 cells were seeded in 24-well plates and infected at 80–90% confluency. An MOI of 100 bacteria was used and infection was carried out for 2 hours, followed by an hour of treatment with 100 μg/ml gentamicin. The fraction of bacteria adhering to the epithelial cell surface was procured prior to gentamicin treatment by performing three washes with PBS, followed by addition of 500μl of 0.25% trypsin-EDTA and 1% triton X-100 each. Bacterial cell suspensions thus obtained were suitably diluted in PBS and plated on LB agar for CFU counts. Similarly, the intracellular bacterial counts from within PD07i and IMCD3 were obtained post gentamicin treatment and suitable dilutions of bacterial suspensions were plated on LB agar for CFU counts.

### Hfq co-IP and RNA-seq data analysis

UTI89Hfq 3xFLAG cultured overnight in LB medium was used to infect PD07i bladder epithelial cells and harvested 3hrs post infection. In parallel, UTI89Hfq 3xFLAG cultured overnight in LB medium was grown statically in Epilife cell culture medium and maintained at 37°C and 5% CO_2_ for the duration of infection to serve as the reference condition. 10^8^ bacterial cells were harvested in IP buffer (10 mM Tris pH 7.5, 150 mM KCl, 1 mM MgCl_2_, Protease inhibitor and Ambion AntiRNAse) and lysed by French press. Cell lysate was incubated with 100 μl of M2 paramagnetic anti-FLAG agarose beads (Sigma) that had been pre-washed twice in IP buffer, for 4 hours at 4°C. Beads were washed twice with cold IP buffer and phenol-chloroform extraction of RNA was performed. Downstream processing of purified co-IP RNA was performed and sequencing was carried out on an Illumina HiSeq2000 platform by Vertis Biotechnology (Munich, Germany).

The quality of raw cDNA reads was examined using FastQ and barcodes clipped. Sequence reads (50 nt) were mapped to UTI89 genome (NC_007946.1) and visualized using the Integrated Genome Viewer (Broad Institute). Read counts were calculated using SeqMonk (Babraham Bioinformatics) by normalizing for total reads mapping to the genome in each cDNA library and gene length. Exact duplicate reads were rejected and gene-wise quantitation was performed by counting the number of reads with a minimum of 10 nt overlap to gene annotations. SIPHT [39] programme was used to predict candidate sRNAs within the UTI89 genome and these predictions were compared to RNA-seq data to find novel candidate sRNAs.

### Agglutination assays

Cultures were grown statically overnight in LB at 37°C. A 10 mg/ml suspension of bakers’ yeast in PBS was freshly prepared for yeast agglutination and fresh 8% erythrocyte suspension from type A- whole blood was used for hemagglutination. Equal volumes of the overnight bacterial culture was mixed with the yeast/erythrocyte suspensions on a glass slide and observed for visible agglutination with and without addition of freshly prepared 3% α-D-mannose.

### Northern blotting and primer extension

Total RNA was isolated using heavy phase lock gel tubes (5Prime) and Northern blot analysis of *papR* (JMJ834), *C271* (JMJ767) and 5S was performed as described previously [88]. Samples were electrophoresed along with a RiboRuler low range RNA ladder (Thermo Scientific) and transferred onto Zeta-probe membranes (Bio-Rad). Membranes probed with radiolabelled primers were visualized using a Typhoon FLA9500 scanner (GE Healthcare) and bands quantified where appropriate using Image Studio Lite Version 4.0 software. Primer extension analysis was carried out as described by Franch *et al*.[89] using primers JMJ832+JMJ833 to amplify PapR with upstream and downstream flanking regions by PCR to serve as template for the sequencing reaction and radiolabelled JMJ834 was used in both sequencing and reverse transcription reactions. Relevant primers are listed in S3 Table.

### Western blotting

~10^8^ bacteria were harvested from appropriate cultures by spinning down cells equivalent to OD_600_ = 1. Bacterial pellets were then resuspended in 1× SDS loading buffer (60 mm Tris-HCl, pH 6.8, 2% SDS, 10% glycerol, 0.005% bromophenol blue, 5 mm EDTA, 0.1 m DTT), boiled for 5 min and separated on a 4–12% Invitrogen NuPage novex Bis-Tris mini gels as detailed by Boysen *et*.*al* [87]. The proteins transferred onto PVDF membranes (Amersham Hybond) were hybridized with 1:20000 dilution of primary anti-GFP (Roche), 1:2000 anti-Fim, 1:1000 anti-PapA and a loading control of 1:50000 dilutions of primary anti-GroEL as appropriate. 1:2000 dilutions of appropriate secondary antibodies (Dako, Denmark) were used in the SNAP i.d.2.0 blotting system (Millipore). Chemiluminescent visualization of protein bands was performed using the Thermo Scientific Pierce ECL Western Blotting Substrate according to the manufacturer’s instructions.

### Flow cytometry and immunofluorescence microscopy

Specimens for flow cytometry and immunofluorescence microscopy were fixed with 3.7% paraformaldehyde for 10 min. and washed gently with PBS three times. 2% BSA for 10 min. was used to block nonspecific signals after which samples were washed once with PBS and stored at 4°C overnight. Bacterial strains were stained with 1:500 anti-Fim [90] and 1:500 anti-PapA followed by 1:200 secondary antibody anti-rabbit conjugated with Alexafluor488 for 1 hour each. All solutions were prepared fresh on the day. Anti-Fim and anti-PapA primary polyclonal antibodies were generated against the entire extracellular type 1 fimbrial filament and PapA respectively. Flow cytometry was performed on a BD FACSAria II flow cytometer (Becton, Dickinson and Company). Data from 10^5^ events per sample was collected and analyzed using BD FACSDiva software (Becton, Dickinson and Company). Strains were distinguished first on the basis of forward scatter area (FSC-A) and side scatter area (SSC-A) and second on the basis of forward scatter area (FSC-A) and FITC fluorescence area (FITC-A). Microscopy was performed using a Leica DMRE fluorescence microscope. Image contrast and addition of scale bars were performed using ImageJ software.

### β-galactosidase activity measurement

Bacteria were resuspended in PBS at appropriate concentrations and 100 μl was used along with 900 μl of Z-buffer (1 M Na_2_CO_3_, 0.27% β-mercaptoethanol) for each replicate in the assay. Samples were incubated with 4 mg/ml ONPG (ortho-Nitrophenyl-β-galactoside) to allow development of yellow colour and reactions were terminated with addition of 1 M Na_2_CO_3_. Optical density was measured at wavelengths of 550 nm and 420 nm and Miller units calculated.

### Statistical analysis

All infection, β-galactosidase assays and flow cytometry experiments were conducted using biological triplicates and mean values and standard deviations were calculated and plotted. Student’s t-test was used to calculate *p-values* and determine statistical significance using GraphPad Prism 5.01 software. *P-values* of less than 0.05 were defined as significant for all experiments.

### Accession number

The RNA-seq data has been deposited in the EBI-ArrayExpress database, Accession number E-MTAB-3498.

## Supporting Information

S1 Fig PapR and C271 sequence conservation. Multiple sequence alignment of (A) PapR and (B) C271 sRNAs showed a high level of sequence and secondary structure conservation among related Gram-negative strains. Alignments were generated using LocARNA software tool [91], complementary base pairs are coloured red and the hue represents sequence conservation.
(TIF)

S2 Fig Mapping C271 to the UTI89 genome. (A) Primer extension was performed to map transcription start site of C271 alongside Sanger sequencing reactions to be read from left to right as TACG. (B) Illustration of the genomic context of C271 in UTI89, drawn to scale.
(TIF)

S3 Fig PapR secondary structure. *In silico* secondary structure prediction of PapR sRNA using Mfold software. The predicted region of interaction between PapR and *papI* mRNA is illustrated in red extending over a single stranded region and the terminal stem-loop. The nucleotide bases in the terminal stem that were inverted in the modified PapR* are denoted by an asterisk (*).
(TIF)

S4 Fig Growth curves. UTI89/pNDM220, UTI89Δ*papR*/pNDM220 and UTI89Δ*papR*/pSK1 were grown at 37°C in aerated LB medium (**−**) and Epilife cell culture medium (−). Optical density was measured at 600 nm wavelength at 60 min intervals and plotted against time in hours. Mean OD_600_ from three independent experiments along with their standard deviations was plotted.
(TIF)

S5 Fig Invasion assay. PD07i bladder cells (grey) and IMCD3 kidney medullary collecting duct cells (red) cultured in 24-well plates were infected with UTI89/pNDM220, UTI89Δ*papR*/pNDM220, UTI89Δ*papR*/pSK1 and UTI89Δ*hfq* for 2 h followed by 1 h of gentamycin treatment to kill extracellular bacteria. Strains were either treated (+) or left untreated (-) with 3% α-D-mannose. Bacterial invasion was assessed by calculating mean CFU counts from three independent experiments and *p-value* (* <0.05) calculated by t-test.
(TIF)

S6 Fig Immunofluorescence imaging and western blotting of fimbriae. UTI89wt stained with anti-Fim (A) and anti-PapA (B) antibodies were examined by immunofluorescence microscopy to demonstrate staining specificity. The panels on the left represent anti-Fim (Ai) and anti-PapA (Bi) fluorescence images and the panels to the right (Aii and Bii) represent the corresponding phase contrast images. Red arrowheads mark unstained UTI89wt and black arrowheads mark UTI89wt cells that stain positive for the particular fimbriae. Scale bars set at 10 μm. Western blots show single bands corresponding to Fim (Aiii) and PapA (Biii). GroEL was used as the internal loading control.
(TIF)

S1 Table Alternate mRNA target predictions for PapR and C271 sRNAs generated using CopRNA (Freiburg RNA tools suite) [41]. (PDF)

S2 Table List of strains used in the study. (PDF)

S3 Table Primers used in this study. (PDF)
