# Supplementary figures and images for: sRNA-Mediated Regulation of P-Fimbriae Phase Variation in Uropathogenic Escherichia coli
Source: PLoS Pathog. 2015 Aug 20;11(8):e1005109. doi: 10.1371/journal.ppat.1005109 (PMC4546395; doi:10.1371/journal.ppat.1005109)

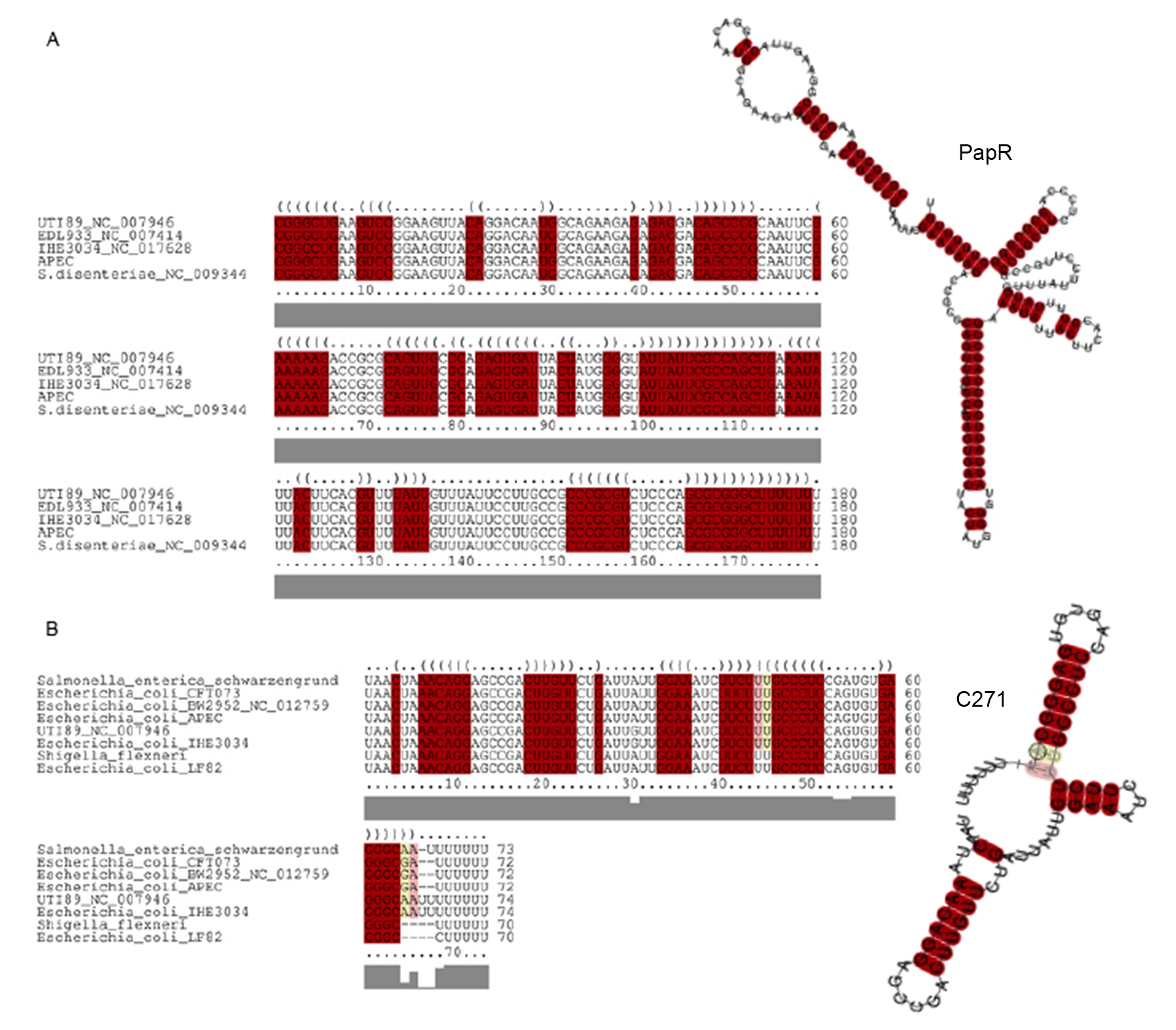

Supplement: S1 Fig — Multiple sequence alignment of (A) PapR and (B) C271 sRNAs showed a high level of sequence and secondary structure conservation among related Gram-negative strains. Alignments were generated using LocARNA software tool [91], complementary base pairs are coloured red and the hue represents sequence conservation. (TIF) [file ppat.1005109.s001.tif]

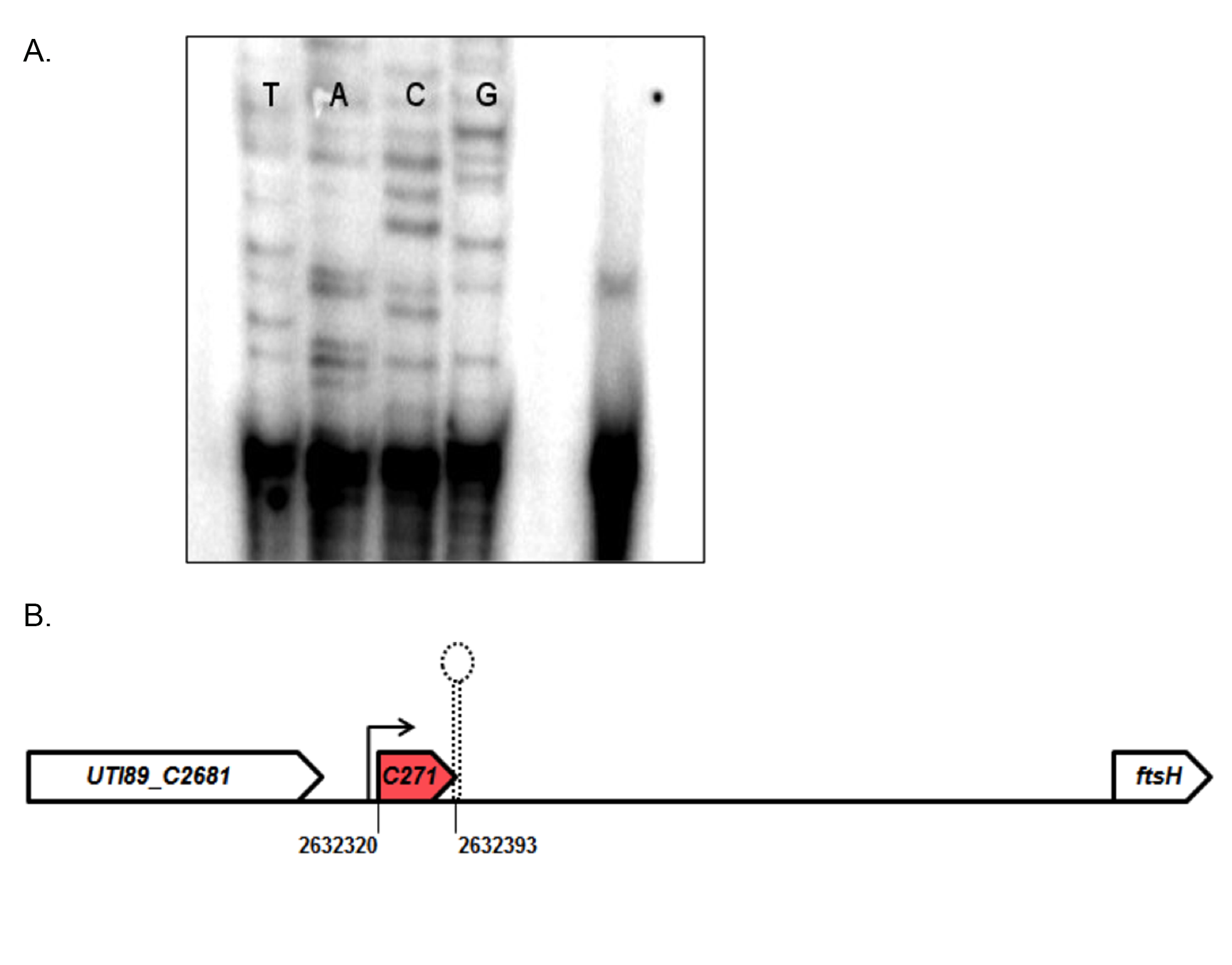

Supplement: S2 Fig — (A) Primer extension was performed to map transcription start site of C271 alongside Sanger sequencing reactions to be read from left to right as TACG. (B) Illustration of the genomic context of C271 in UTI89, drawn to scale. (TIF) [file ppat.1005109.s002.tif]

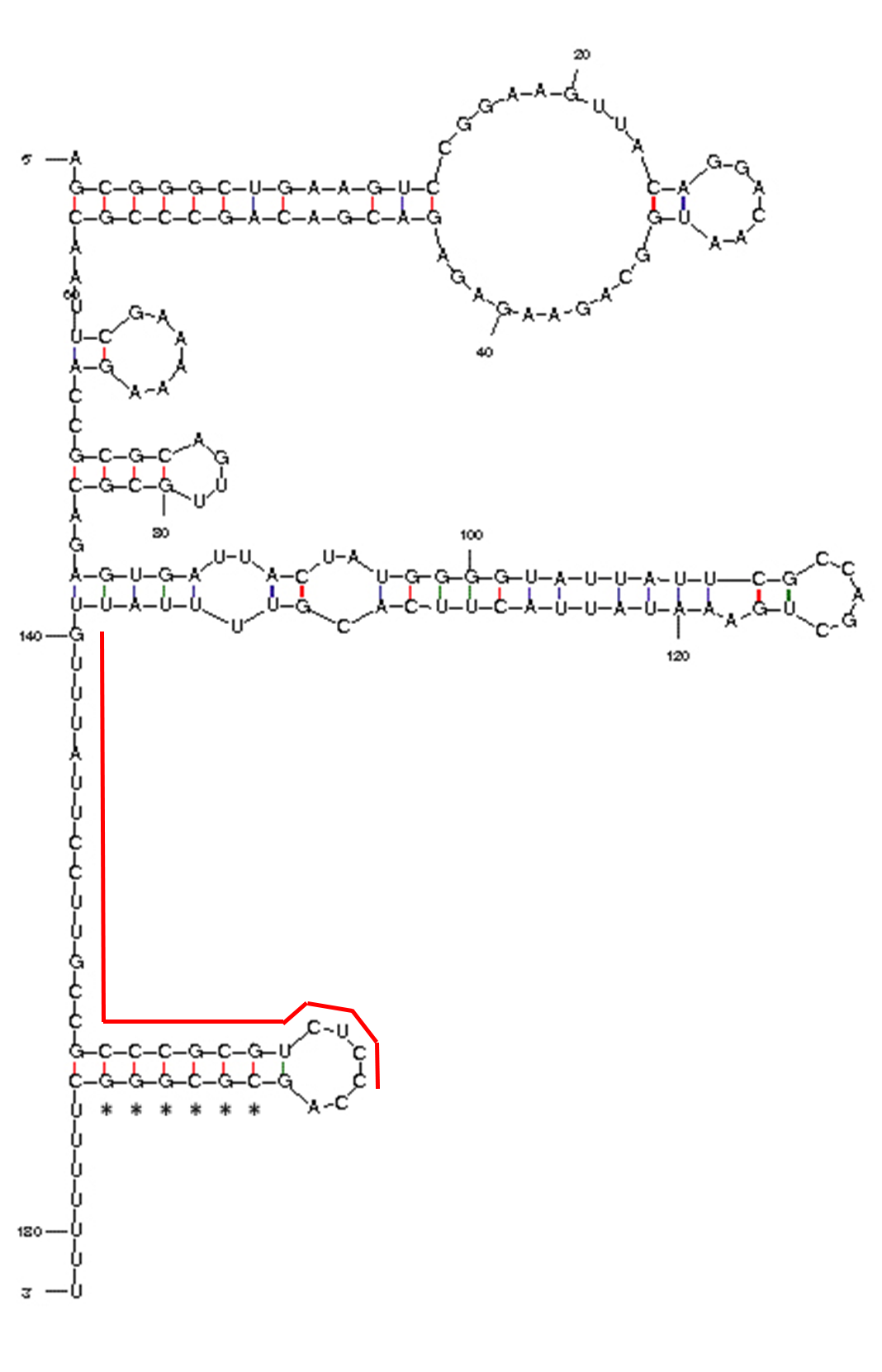

Supplement: S3 Fig — In silico secondary structure prediction of PapR sRNA using Mfold software. The predicted region of interaction between PapR and papI mRNA is illustrated in red extending over a single stranded region and the terminal stem-loop. The nucleotide bases in the terminal stem that were inverted in the modified PapR* are denoted by an asterisk (*). (TIF) [file ppat.1005109.s003.tif]

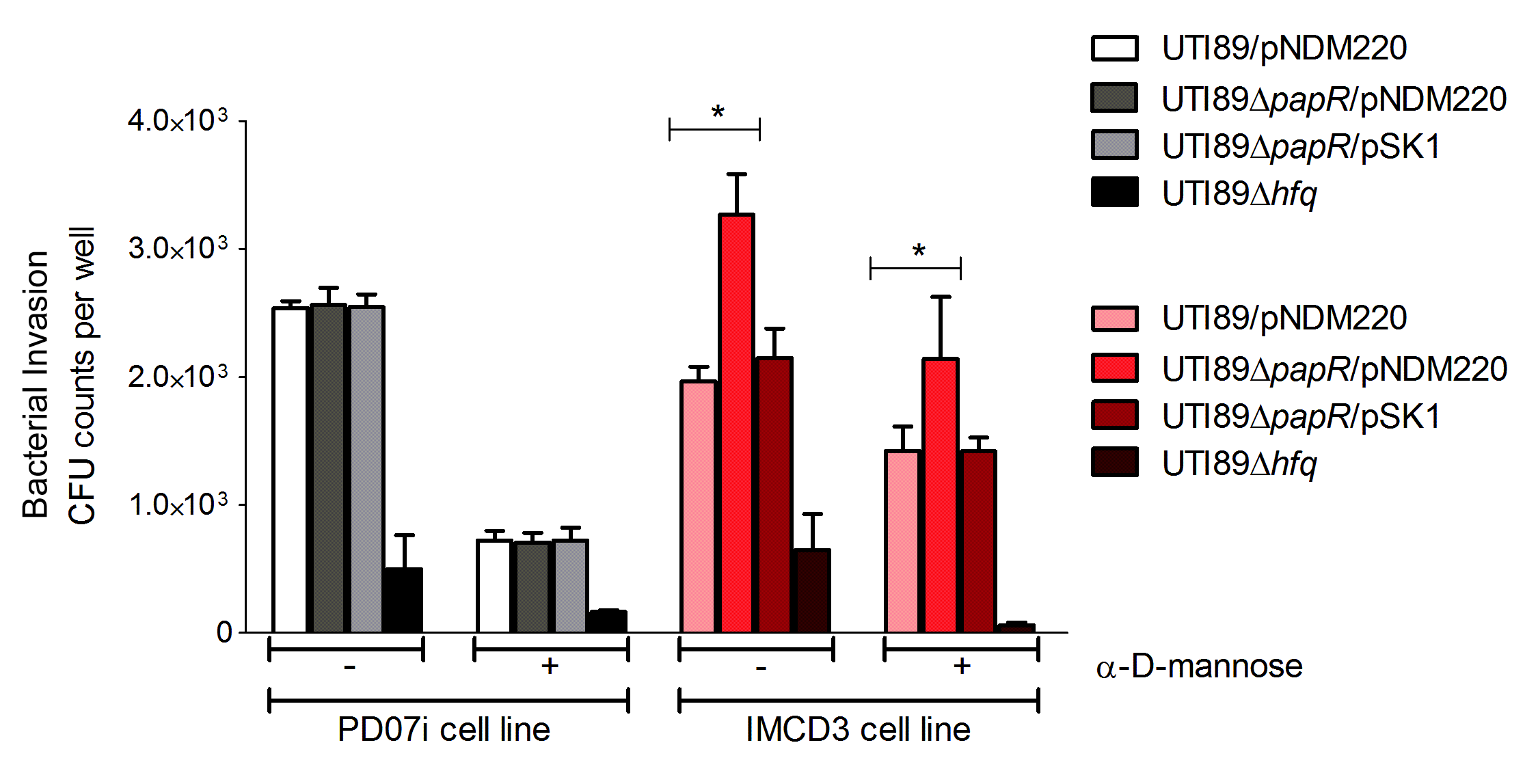

Supplement: S5 Fig — PD07i bladder cells (grey) and IMCD3 kidney medullary collecting duct cells (red) cultured in 24-well plates were infected with UTI89/pNDM220, UTI89ΔpapR/pNDM220, UTI89ΔpapR/pSK1 and UTI89Δhfq for 2 h followed by 1 h of gentamycin treatment to kill extracellular bacteria. Strains were either treated (+) or left untreated (-) with 3% α-D-mannose. Bacterial invasion was assessed by calculating mean CFU counts from three independent experiments and p-value (* <0.05) calculated by t-test. (TIF) [file ppat.1005109.s005.tif]

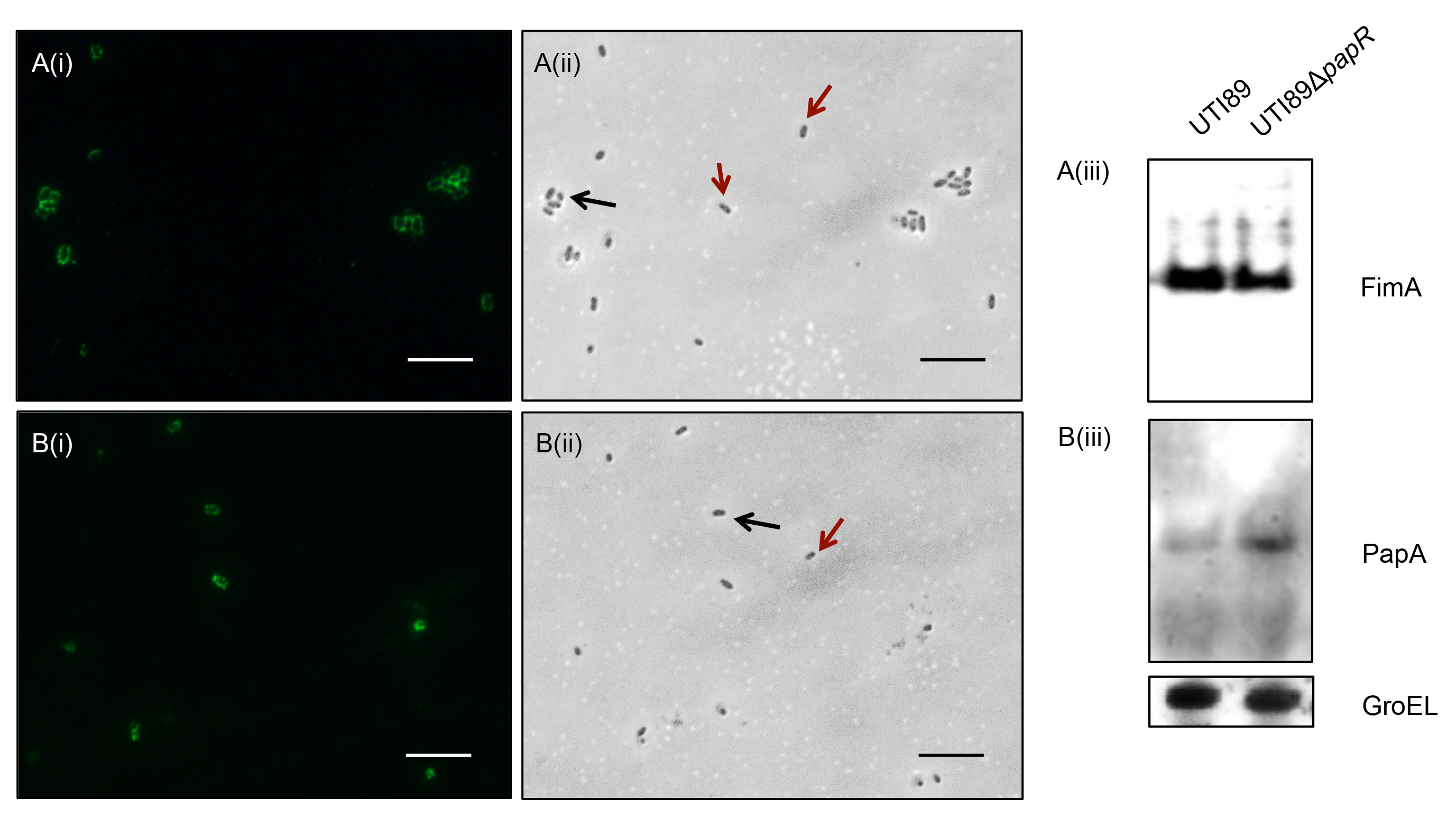

Supplement: S6 Fig — UTI89wt stained with anti-Fim (A) and anti-PapA (B) antibodies were examined by immunofluorescence microscopy to demonstrate staining specificity. The panels on the left represent anti-Fim (Ai) and anti-PapA (Bi) fluorescence images and the panels to the right (Aii and Bii) represent the corresponding phase contrast images. Red arrowheads mark unstained UTI89wt and black arrowheads mark UTI89wt cells that stain positive for the particular fimbriae. Scale bars set at 10 μm. Western blots show single bands corresponding to Fim (Aiii) and PapA (Biii). GroEL was used as the internal loading control. (TIF) [file ppat.1005109.s006.tif]
