# Supplementary material for: sRNA-Mediated Regulation of P-Fimbriae Phase Variation in Uropathogenic Escherichia coli
Source: PLoS Pathog. 2015 Aug 20;11(8):e1005109. doi: 10.1371/journal.ppat.1005109 (PMC4546395; doi:10.1371/journal.ppat.1005109)
Supplement: S1 Table — (PDF) [file ppat.1005109.s007.pdf]

S1 Table

Prediction p-value	Energy [kcal/mol]	Position mRNA	Position sRNA	PapR Target Annotation
1.57E-06	-19.83	216 -- 240	1 -- 26	4-amino-4-deoxychorismate lyase PabC
3.43E-06	-21.13	139 -- 161	14 -- 38	phosphocarrier protein HPr
6.68E-06	-23.16	284 -- 311	138 -- 167	phage capsid protein
7.98E-06	-24.6	14 -- 53	134 -- 165	2-octaprenyl-6-methoxyphenyl hydroxylase UbiH
1.61E-05	-20	36 -- 72	122 -- 164	protein NinE
2.96E-05	-19.51	137 -- 168	138 -- 164	conjugal transfer protein TraI
8.87E-05	-16.89	33 -- 73	121 -- 159	methyltransferase
0.0002105	-13.89	39 -- 91	108 -- 147	osmotically-inducible protein OsmE
0.0002703	-16.34	8 -- 50	108 -- 165	transcriptional regulator MraZ
0.0002744	-18.8	8 -- 43	130 -- 164	leucine-responsive regulatory protein LRP

Prediction p-value	Energy [kcal/mol]	Position mRNA	Position sRNA	C271 Target Annotation
1.58E-07	-15.92	156 -- 189	17 -- 45	DNA polymerase III subunit beta DnaN
3.71E-07	-16.68	108 -- 151	7 -- 40	long-chain fatty acid outer membrane transporter FadL
1.81E-06	-18.26	130 -- 160	11 -- 44	ribosomal RNA small subunit methyltransferase H YgjO
3.55E-06	-17.31	20 -- 46	10 -- 38	UDP-N-acetylmuramoylalanine--D-glutamate ligase MurD
8.38E-06	-15.08	14 -- 70	8 -- 64	ethanol dehydrogenase EutE
2.71E-05	-13.61	184 -- 214	16 -- 45	NUDIX hydrolase YfaO
0.0001518	-13.84	146 -- 167	12 -- 38	multidrug export protein EmrA
0.0002294	-11.87	185 -- 239	9 -- 64	PulS_OutS family protein
0.0002805	-12.77	131 -- 171	9 -- 45	curli production assembly/transport component CsgE
0.0003137	-14.1	6 -- 38	7 -- 43	hydrogen peroxide-inducible genes activator

S1 Table: Alternate mRNA target predictions for PapR and C271 sRNAs generated using CopRNA (Freiburg RNA tools suite) [1]

1. Wright PR, Richter AS, Papenfort K, Mann M, Vogel J, et al. (2013) Comparative genomics boosts target prediction for bacterial small RNAs. Proc Natl Acad Sci U S A 110: E3487-3496.
