# Supplementary material for: sRNA-Mediated Regulation of P-Fimbriae Phase Variation in Uropathogenic Escherichia coli
Source: PLoS Pathog. 2015 Aug 20;11(8):e1005109. doi: 10.1371/journal.ppat.1005109 (PMC4546395; doi:10.1371/journal.ppat.1005109)
Supplement: S2 Table — (PDF) [file ppat.1005109.s008.pdf]

Supplementary Table 2

Strains	Genotype	Source
UTI89	Cystitis-derived isolate, serotype O18:K1:H7	[2]
Top10	F- *mcrA* Δ(*mrr-hsd* RMS-*mcrBC*) φ80*lacZ*ΔM15 Δ*lacX*74 *nupG recA1 araD139* Δ(*ara-leu*)7697 *galE*15 *galK*16 *rpsL*(StrR) endA1 λ-	Invitrogen
UTI89Δ*hfq*	Δ*hfq*, Cml^R	This study
UTI89 Hfq 3xFLAG	3xFLAG *hfq*	This study
UTI89Δ*ripA*	Δ*ripA*, Cml^R	This study
UTI89/pSK1	pNDM220, Mini-R1, bla, $LacI^q$, $P_{A1/O4/O3}$:: *ripA*	This study
UTI89Δ*ripA*/pSK1/pSKpapI	Δ*ripA*, $P_{A1/O4/O3}$:: *ripA*, *papI::gfp*	This study
UTI89Δ*ripA*/pSK1*/pSKpapI	Δ*ripA*, $P_{A1/O4/O3}$:: *ripA**, *papI::gfp*	This study
UTI89Δ*lrp*	Δ*lrp*, Kan^R	This study
DL1504	MC4100 λ354 lysogen Kan^R	Bernt Eric Uhlin
DL1504/pSK1	pNDM220, Mini-R1, bla, $LacI^q$, $P_{A1/O4/O3}$:: *ripA*	This study
DL1504Δ*papI*	MC4100 λ354 lysogen Kan^R, ΔpapI	Bernt Eric Uhlin
DL1504Δ*papI*/pSK1	pNDM220, Mini-R1, bla, $LacI^q$, $P_{A1/O4/O3}$:: *ripA*	This study
DL2121	MC4100 λ354-13 lysogen Kan^R	Bernt Eric Uhlin
DL2121/pSK1	pNDM220, Mini-R1, bla, $LacI^q$, $P_{A1/O4/O3}$:: *ripA*	This study
UTI89Δ*lrp*/pSKlrp	Δ*lrp*, pNDM220, Mini-R1, bla, $LacI^q$, $P_{A1/O4/O3}$:: *lrp*	This study
UTI89Δ*hfq*/pJMJ220	Δ*hfq*, pNDM220, Mini-R1, bla, $LacI^q$, $P_{A1/O4/O3}$:: *hfq*	This study
**Plasmids**	**Genotype**	
pKD3	Cml, template plasmid	[3]
pKD4	Kan, template plasmid	
pKD46	Bla, λ Red recombinase expression plasmid	
pNDM220	Mini-R1, bla, $LacI^q$, $P_{A1/O4/O3}$	[4]
pSUB11	3xFLAG FRT ahp FRT bla R6KoriV	[5]
pXG10	pSC101 derivative, constitutive P_{LtetO} promoter	[6]
pJMJ220	pNDM220, Mini-R1, bla, $LacI^q$, $P_{A1/O4/O3}$:: *hfq*	[7]

References:

1. Smith, C., et al., *Freiburg RNA Tools: a web server integrating INTARNA, EXPARNA and LOCARNA.* Nucleic Acids Res, 2010. **38**(Web Server issue): p. W373-7.
2. Mulvey, M.A., J.D. Schilling, and S.J. Hultgren, *Establishment of a persistent Escherichia coli reservoir during the acute phase of a bladder infection.* Infect Immun, 2001. **69**(7): p. 4572-9.
3. Datsenko, K.A. and B.L. Wanner, *One-step inactivation of chromosomal genes in Escherichia coli K-12 using PCR products.* Proc Natl Acad Sci U S A, 2000. **97**(12): p. 6640-5.
4. Gotfredsen, M. and K. Gerdes, *The Escherichia coli relBE genes belong to a new toxin-antitoxin gene family.* Mol Microbiol, 1998. **29**(4): p. 1065-76.
5. Uzzau, S., et al., *Epitope tagging of chromosomal genes in Salmonella.* Proc Natl Acad Sci U S A, 2001. **98**(26): p. 15264-9.
6. Urban, J.H. and J. Vogel, *Translational control and target recognition by Escherichia coli small RNAs in vivo.* Nucleic Acids Res, 2007. **35**(3): p. 1018-37.
7. Simonsen, K.T., et al., *A role for the RNA chaperone Hfq in controlling adherent-invasive Escherichia coli colonization and virulence.* PLoS One, 2011. **6**(1): p. e16387.
