# Supplementary material for: sRNA-Mediated Regulation of P-Fimbriae Phase Variation in Uropathogenic Escherichia coli
Source: PLoS Pathog. 2015 Aug 20;11(8):e1005109. doi: 10.1371/journal.ppat.1005109 (PMC4546395; doi:10.1371/journal.ppat.1005109)
Supplement: S3 Table — (PDF) [file ppat.1005109.s009.pdf]

S3 Table

Primer	Description	Sequence
JMJ63	Δ*hfq*_KO_F	ATGGCTAAGGGGCAATCTTTACAAGATCCGTTCCTGAACGCACTG CGTCGGTGTAGGCTGGAGCTGCTTCG
JMJ64	Δ*hfq*_KO_R	TTATTCGGTTTCTTCGCTGTCCTGTTGCGCGGAAGTATTCTGCGCG CTGCCATATGAATATCCTCCTTAGTTCC
JMJ754	Δ*ripA*_KO_F	CGGGATAATATTGTGAGTAAAATAAGGAGCGGGCTGAAGTCCGGA AGTTACAGGACAATGGCAGAAGAGAGTGTAGGCTGGAGCTGCTT C
JMJ755	Δ*ripA*_KO_R	GATACGCGGCTTTAGCCCCTCTCCGGAGGGGTTTTCTTGTGGGCA AAAAAAGCCCGCGCTGGGAGACGCGCATATGAATATCCTCCTTA
JMJ649	Δ*lrp*_KO_F	TGAACAGTGATGTTTCAGGGTCAGACAGGAGTAGGGAAGGAATA CAGAGAGACAATAATAATGGTAGTGTAGGCTGGAGCTGCTTCG
JMJ650	Δ*lrp*_KO_R	CCGTGTTAGCGCGTCTTAATAACCAGACGATTACTCTGCTTGACTT CTTCCATAACAACGCATATGAATATCCTCCTTAGTTCC
JMJ71	Kan_F	CGGTGCCCTGAATGAACTGC
JMJ72	Kan_R	CGGTGCCCTGAATGAACTGC
JMJ99	Cml_F	TTATACGCAAGGCGACAAGG
JMJ100	Cml_R	GATCTTCCGTCACAGGTAGG
JMJ155	Hfq 3xFLAG_F	CAGCAGTAACTACCATCATGGTAGCAGCGCGCAGAATACTTCCGC GCAACAGGACAGCGAAGAAACCGAAGACTACAAAGACCATGAC GGTGATTATAAA
JMJ156	Hfq 3xFLAG_R	TAACCCTCTAAATAGATCAGCGGGGAACGCAGGATCGCTGGCTCC CCGTGTAAAAAAACAGCCCGAAACCCATATGAATATCCTCCTTAG
JMJ835	pSK1_F	GCCTGACGTCGGCAAAAAGAGTGTTGACTTGTGAGCGGATAACA ATGATACTTAGATTCCGGGCTGAAGTCCGGAAGTT
JMJ836	pSK1_R	CCCCGGATCCAAAAAAAGCCCGCGCTGGGAGA
JMJ645	pSK1*_R	CCCCGGATCCAAAAAAACGGGCGCATGGGAGCGCGCCCGGGCA AGGAATAAACAATAAAACGTGAAG
JMJ939	pSKlrp_F	CCCCCCGACGTCGGCAAAAAGAGTGTTGACTTGTGAGCGGATAA CAATGATACTTAGATTCAATCTTCATGATTTCGGTCT
JMJ940	pSKlrp_R	CCCCGGATCCTTAGCGCGTCTTAATAACCAGACGA
JMJ207	pNDM220_F	GGCCTCTTCGCTATTACGCG
JMJ221	pNDM220_R	TTGTCTCATGAGCGGATACA
JMJ641	pSKpapI_F	CCCCATGCATGAAGAAACTTTGTTACAGTGAG
JMJ642	pSKpapI_R	CCCCGCTAGCAGTTGTGGAAGAACAGTTTTGTC
JMJ732	pXG10_F	TGGGATATATCAACGGTGGT
JMJ733	pXG10_R	CCGTATGTAGCATCACCTTC
JMJ767	RipB_NB	AAAGAAGATTTCCAACAATCAGAACAAGTCGGC
JMJ832	RipA_pxt_F	TGTTTAAATGCCATGAATCCTC
JMJ833	RipA_pxt_R	GCGGCAAGGAATAAACAATAAA
JMJ834	RipA_NB	CGCGGTCTTTTTCGAATTGCGGGCTGTCGTCTCTC
5S		CGGCGCTACGGCGTTTCACTTCTG
